# Case Report: Abdominal Lymph Node Metastases of Parathyroid Carcinoma: Diagnostic Workup, Molecular Diagnosis, and Clinical Management

**DOI:** 10.3389/fendo.2021.643328

**Published:** 2021-03-23

**Authors:** Christina Lenschow, Carmina Teresa Fuss, Stefan Kircher, Andreas Buck, Ralph Kickuth, Joachim Reibetanz, Armin Wiegering, Albrecht Stenzinger, Daniel Hübschmann, Christoph Thomas Germer, Martin Fassnacht, Stefan Fröhling, Nicolas Schlegel, Matthias Kroiss

**Affiliations:** ^1^ Department of General, Visceral, Transplant, Vascular and Pediatric Surgery, University Hospital Würzburg, University of Würzburg, Würzburg, Germany; ^2^ Department of Internal Medicine I, Division of Endocrinology and Diabetes, University Hospital, University of Würzburg, Würzburg, Germany; ^3^ Institute of Pathology, University of Würzburg, Würzburg, Germany; ^4^ Department of Nuclear Medicine, University Hospital Würzburg, University of Würzburg, Würzburg, Germany; ^5^ Department of Diagnostic and Interventional Radiology, University Hospital Würzburg, University of Würzburg, Würzburg, Germany; ^6^ Institute of Pathology, University Hospital Heidelberg, Heidelberg, Germany; ^7^ Germany and German Cancer Consortium (DKTK), Heidelberg Partner Site, Heidelberg, Germany; ^8^ Computational Oncology, Molecular Diagnostics Program, NCT Heidelberg and Heidelberg University Hospital, Heidelberg, Germany; ^9^ Comprehensive Cancer Center Mainfranken, University of Würzburg, Würzburg, Germany; ^10^ National Center for Tumor Diseases (NCT Heidelberg), Division of Translational Medical Oncology German Cancer Research Center (DKFZ), University Hospital Heidelberg, Heidelberg, Germany; ^11^ Department of Medicine IV, University Hospital Munich, Ludwig-Maximilians-Universität München, Munich, Germany

**Keywords:** parathyroid carcinoma, abdominal lymph node metastases, molecular diagnostics, repeated surgery, [18F]FDG-PET-CT, immune check inhibitor, pembrolizumab

## Abstract

Parathyroid carcinoma (PC) is an orphan malignancy accounting for only ~1% of all cases with primary hyperparathyroidism. The localization of recurrent PC is of critical importance and can be exceedingly difficult to diagnose and sometimes futile when common sites of recurrence in the neck and chest cannot be confirmed. Here, we present the diagnostic workup, molecular analysis and multimodal therapy of a 46-year old woman with the extraordinary manifestation of abdominal lymph node metastases 12 years after primary diagnosis of PC. The patient was referred to our endocrine tumor center in 2016 with the aim to localize the tumor causative of symptomatic biochemical recurrence. In view of the extensive previous workup we decided to perform [18F]FDG-PET-CT. A pathological lymph node in the liver hilus showed slightly increased FDG-uptake and hence was suspected as site of recurrence. Selective venous sampling confirmed increased parathyroid hormone concentration in liver veins. Abdominal lymph node metastasis was resected and histopathological examination confirmed PC. Within four months, the patient experienced biochemical recurrence and based on high tumor mutational burden detected in the surgical specimen by whole exome sequencing the patient received immunotherapy with pembrolizumab that led to a biochemical response. Subsequent to disease progression repeated abdominal lymph node resection was performed in 10/2018, 01/2019 and in 01/2020. Up to now (12/2020) the patient is biochemically free of disease. In conclusion, a multimodal diagnostic approach and therapy in an interdisciplinary setting is needed for patients with rare endocrine tumors. Molecular analyses may inform additional treatment options including checkpoint inhibitors such as pembrolizumab.

## Introduction

Parathyroid carcinoma PC is an orphan malignancy occurring in approximately 1%–5% (United States, Europe, Japan) of all patients with primary hyperparathyroidism (pHPT) (1–5). The main pre-operative challenge of PC is to raise the suspicion of malignant disease at diagnosis since clinical outcome and prognosis are largely dependent on the extent of primary surgery. Despite the combination of multiple diagnostic modalities, this rare tumor is often difficult to diagnose preoperatively (6–9). Moreover, diagnosis of malignancy is made in only 15% of the fast-frozen sections. So, in the vast majority of cases, only the final histology or a relapse provides the diagnosis (10).

The high rate of relapse is another considerable problem in PC patients. We have recently published a comprehensive clinical characterization of 83 PC cases and have demonstrated that within a median interval of 48 months 38.6% of cases relapsed (7). However, in case of biochemical recurrence, the precise localization of cancerous tissue is mandatory to enable surgical treatment. The calcimimetic drug cinacalcet is approved to control serum calcium and may be used in case of unsuccessful localization and/or advanced, non-resectable disease. Systemic antitumoral therapy has remained anecdotal (11).

To date, only very few cases of PC with abdominal tumor localization (peripancreatic lymph nodes: n=1, liver n= 6) have been described (12–14).

Here, we present the complex diagnostic workup and multimodal therapy in a 46-year old woman with the uncommon manifestation of abdominal metastases of PC.

## Case Description

A 46-year old woman was referred to our endocrine tumor center in 2016 with the aim to localize the tumor causative of biochemical recurrence.

Primary surgery due to pHPT had been performed in 2004. Intraoperatively, PC was suspected and en-bloc-resection (hemithyroidectomy, parathyroidectomy and central lymph node dissection of the left side) was performed. PC was confirmed histopathologically and resection margins were free of tumor (R0). The patient experienced a permanent recurrent laryngeal nerve palsy at the left side as complication of the surgical procedure. She underwent postoperative adjuvant external beam radiation of the thyroid region at a total dose of 50.4 Gy. The patient was subsequently free of disease for 12 years.

In 2016, the patient experienced symptoms similar to those at initial diagnosis with thirst, fatigue and visual flashes. Serum calcium was elevated up to 3.4 mmol/L [reference range: 2.0–2.7mmol/L] and parathyroid hormone (PTH) was increased to 203 pg/mL [10–65pg/ml]. Medication with cinacalcet was initiated. Within the three subsequent months, the patient underwent cervical ultrasonography, CT and MRI of the neck and chest, [^99m^Tc] Sestamibi-SPECT/CT, as well as [11C]methionine-PET-CT. None of these imaging modalities localized tumor recurrence. Additionally, the patient underwent re-exploration of the right neck and lymph node dissection of the right cervical lymph nodes. Histopathology was negative for PC and hypercalcemia persisted postoperatively.

**Figure 1:**
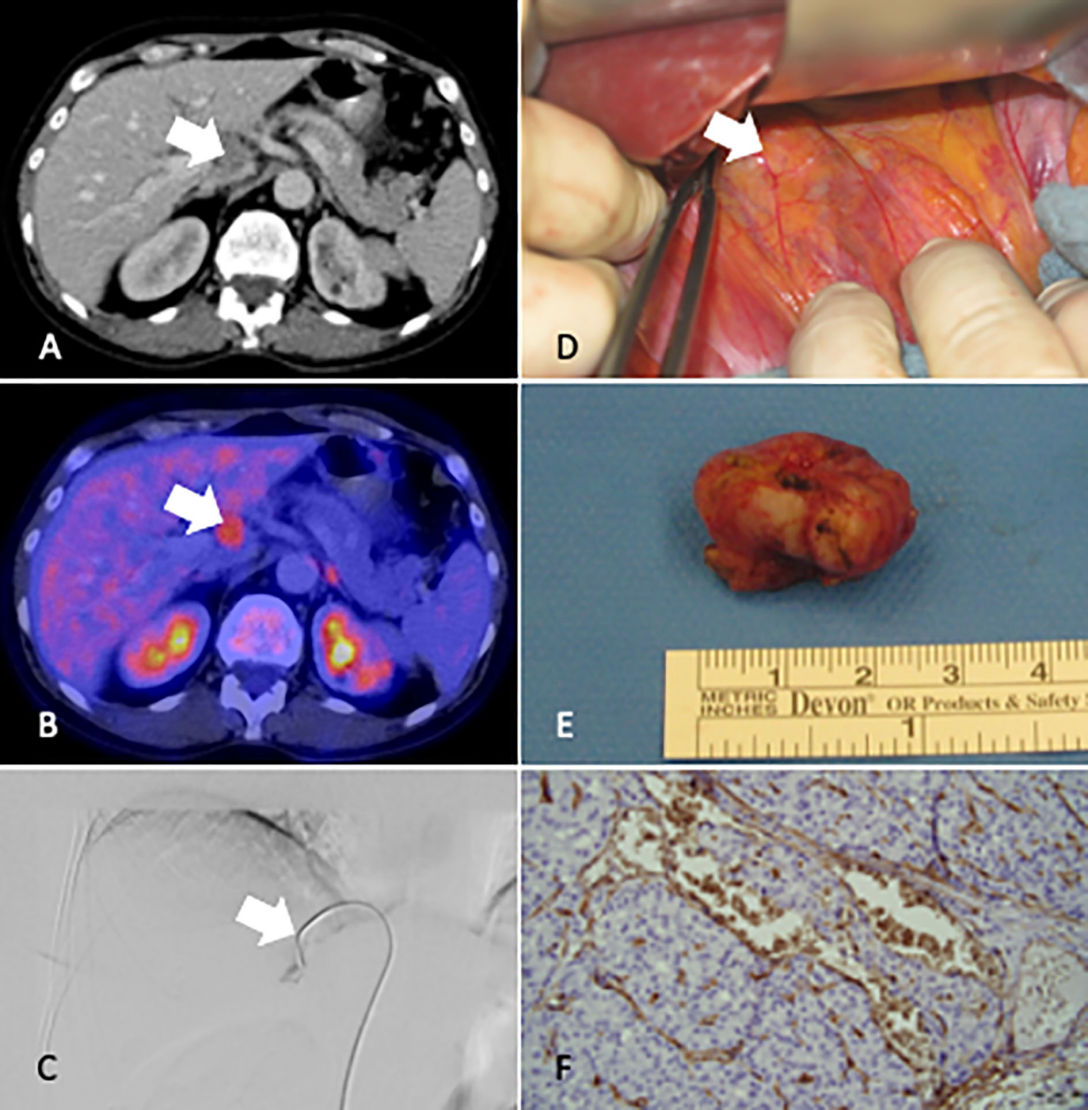
CT Imaging **(A)**, [18F]FDG-PET-CT **(B)**, Venous sampling V. hepatica **(C)**, intraoperative localization **(D)**, Resected lymph node **(E)**, histological result at time of diagnosis recurrence **(F)**, White arrow marks the lymph node metastases in the hilus of the liver.

Subsequently the patient presented herself at our institution. In view of the extensive previous workup we decided to perform [18F]FDG-PET-CT. Surprisingly, a pathological lymph node (17x24 mm) within the liver hilus showed slightly increased FDG-uptake (**Figures 1A, B**). Due to the unusual localization we questioned relationship with the recurrence of PC and performed selective PTH venous sampling that included the entire neck region, but also sampling from the liver veins in addition to V. cava inferior sampling (**Figure 1C**). Highest PTH was measured in the right V. hepatica with 758 pg/ml and a ratio of 1.3 compared to V. cava inferior. After laparotomy and preparation of the liver hilus, the lymph node was resected with intact capsule without any signs of infiltration (R0) in January 2017. Inspection revealed no further lymph node manifestations (**Figures 1D, E**). Intraoperative PTH dropped from 841 ng/L to 387ng/L The peri-and postoperative course was without any complications. The histological assessment of the resected tissue confirmed lymph node metastasis with blood vessel infiltration (V1) of PC (**Figure 1F**). Histological and immunohistochemical analyses were both consistent with parathyroid carcinoma (positivity for PTH, loss of parafibromin). Furthermore, we stained the lymph node sample for PD-L1 (antibody 28-8). In the tumor cell there was no specific membrane-bound positivity (TC Score 0; Cologne -Score for NSCLS) (15). No tumor associated PD-L1 positivity in the background inflammatory infiltrate was observed. Moreover, we analyzed the DNA-Mismatch-Repair-Protein MLH1, MSH2, MSHG, and PMS2. The results showed no evidence of microsatellite instability. In summary, this was a lymph node metastases of a PD-L1-negative, microsatellite stable parathyroid carcinoma.

After resection, Calcium decreased steadily from 3.4 mmol/L to 2.3 mmol/L. Cinacalcet was discontinued.

## Systemic Treatment

Unfortunately, few days after discharge, PTH level and Calcium level increased slowly again requiring the use of cinacalcet. The medication was increased to a daily cumulative dose of 150 mg in the following weeks. Four months after resection, PTH rose to 912 ng/L and additionally, denosumab was required to adequately control serum calcium levels (2.6 mmol/l).

**Figure 2:**
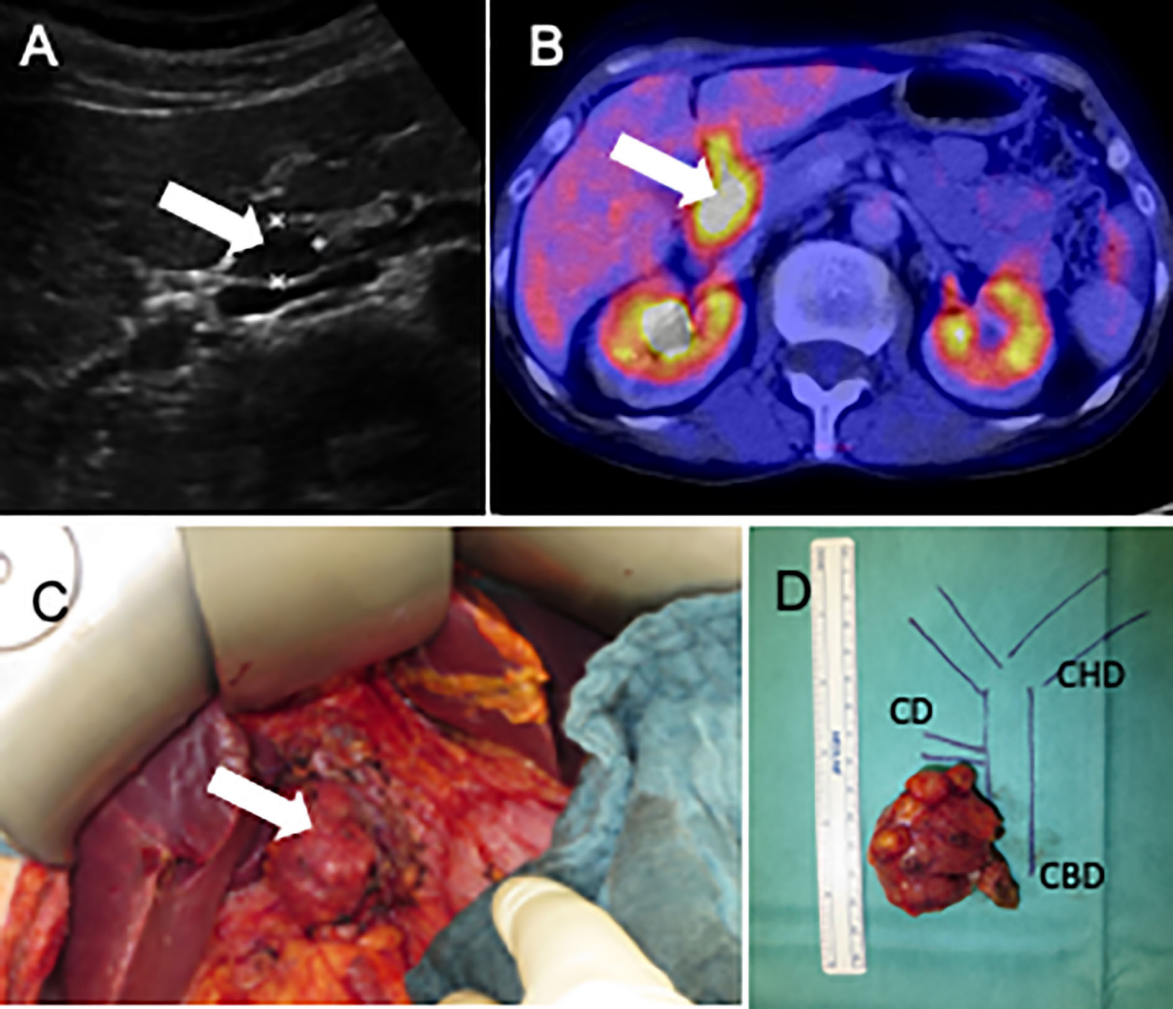
Abdominal ultrasonography **(A)** [18F]FDG-PET-CT **(B)**, photography surgery 10/2018, Lymph node metastases *in situ* **(C)** (white arrows mark the tumor), and the tumor localization in an anatomical drawing **(D)**. CD, cystic duct; CBD, common bile duct; CHD, common hepatic duct.

Abdominal ultrasonography indicated recurrent lymph node metastases in the hepatic hilus (**Figure 2A**) which was confirmed by [18F]FDG-PET-CT.

The patient was included in NCT/DKTK MASTER (Molecularly Aided Stratification for Tumor Eradication Research), a multicenter, prospective observational study that is based on a common workflow for diagnostics, therapeutic decision making, and structured follow-up in patients with rare tumors failing standard treatment. The formalin-fixed paraffin embedded lymph node tissue obtained at surgery was utilized for whole exome sequencing and 412 single nucleotide variants (SNV) and 2 insertions/deletions (indels) were identified.

**Figure 3:**
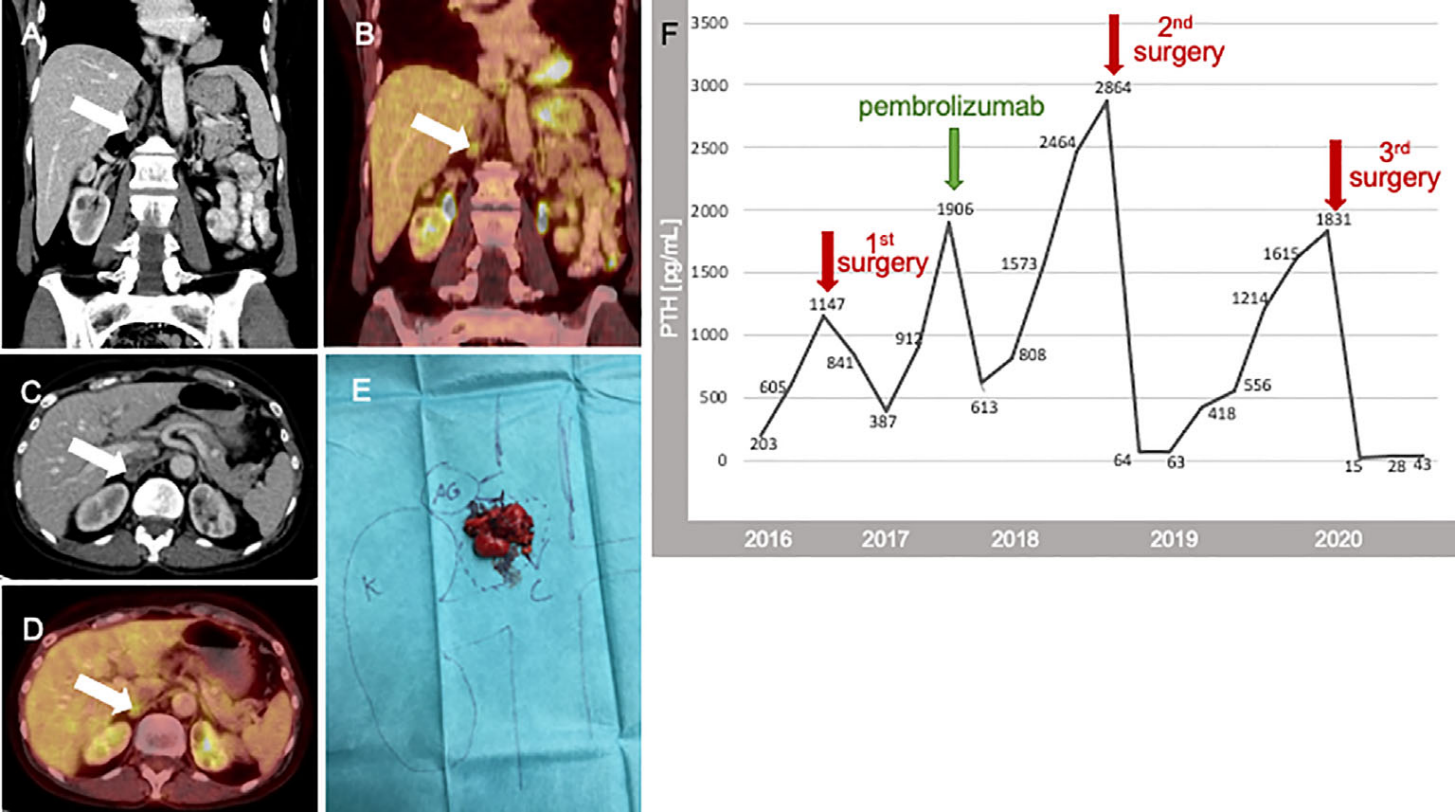
CT Imaging **(A, C)**, 12/2019), [18F]FDG-PET-CT **(B, D)**, (white arrows mark the tumor), photography surgery 01/2020 **E**; K, Kidney; VC, Vena cava; AG, adrenal gland; PTH course is shown as time scale from the beginning of treatment in our hospital 2016 up to 2020. In this period, we performed three abdominal surgeries (red arrow) and the beginning of therapy within Pembrolizumab (green arrow) **(F)**.

Because of the rapid recurrence in the same location, the fact that PTH levels had not returned to normal levels after resection of the previous metastases and in view of controlled serum calcium (2.6 mmol/L) only with 150 mg cinacalcet, we decided to initiate therapy with pembrolizumab on a compassionate use basis at 200 mg every three weeks. Pembrolizumab treatment was started in 09/2017 at a PTH serum concentration of 1,906 ng/L (**Figure 3F**). After four cycles of therapy, PTH dropped significantly (11/2017) to 613 ng/L and [18F]FDG-PET-CT showed stable disease four months later. Pembrolizumab was continued for six infusions every three weeks until [18F]FDG-PET-CT detected a lymph node bulk adjacent to the gallbladder (**Figure 2B**) in 07/2018. After evaluation of resectability, re-laparotomy was performed (10/2018) and the lymph node conglomerate of 3.4x2.0x3.2cm in lymph node station 12 was resected. There were no clinical signs of extra nodal tumor infiltration (**Figures 2C, D**). Intraoperative PTH dropped from 2864 ng/L to 64.3ng/L. Histology confirmed a conglomerate of lymph node metastases of the PC up to 3.8 cm in size exhibiting the same features as the initial specimen. The postoperative course of the patient was unremarkable. In the following months, the patient’s serum calcium and PTH levels increased slowly. The patient was normocalcaemic on intermittent medication with denosumab and had stable PTH levels between 197 und 332 pg/L until 06/2019. At this time a progress of increased serum calcium levels (2.8 mmol/L) and PTH (418 ng/L) was detected. A [18F]FDG-PET-CT was performed and showed a new lymph node recurrence near the diaphragm (**Figures 3A–D**). This lymph node was resected as well as a lymph node dorsal of the V. cava inferior in 01/2020 (**Figure 3E**). PTH dropped intraoperatively to 15.8 ng/L (preoperative: 1,831 ng/L). Due to postoperative hypocalcemia, the patient received decostriol (1,25-dihydroxycholecalciferol) at a dose of 0.5 µg twice a day and up to 3 g calcium P.O. per day which could be discontinued during the following 8 weeks. Until 10/2020 the patient was biochemically free of recurrence (serum calcium 2.4 mmol/L, PTH 42.7 pg/ml) without any medication. The whole course of PTH is shown in **Figure 3F**. Interdisciplinary discussion recommended watchful waiting and restaging in case of biochemical progression.

## Discussion

The diagnostic workup and site directed therapy in case of recurrent PC remains challenging. Distant metastases and especially abdominal localization of PC, as presented in this case report, are highly uncommon (7). The review of the literature revealed case reports of distant metastases, specifically abdominal and brain metastases (12–14) (**Table 1**). Interdisciplinary management is crucial to enable focused surgical treatment and adequate medical treatment (16). In this case report, we presented an example for an interdisciplinary workflow in a patient with abdominal recurrent PC 12 years after primary diagnosis.

**Table 1:** Unusual localizations of distant PC recurrence in the literature.

**First Authors**	**Publication** **Year**	**Patients** **(n=)**	**Sites of Distant Metastases (DM)**	**Finding Modality** **of DM**	**Treatment of DM**	**Outcome**	**Follow up** **(years)**
Manente et al.	1987	1	Lymph node Pancreas region	Not Reported	Resection	Death	1
Qiu Zhong-ling et al.	2013	5	Liver	CT n=5	RFA /EB n=1	Alive n=3	0.5-9
				Biopsy n= 3	PR n=3	Death n=2	
				^99m^TC-MIBI n=1	Unknown=1		
		3	Brain	MRI n=2	PR	Alive n= 2	5.5-9
				CT n=1		Unknown n=1	2.5
Tsoli et al.	2017	1	Brain	MRI	Resection, RTx	Death	2
Asare et al.	2019	2	Liver	Unkown	CTx	Alive n=1	Unkown
						Death n=1	n=6

Distant metastases were only considered, if they were localized extracervical, extrathoracic and were not localized in bones. RFA, radiofrequency ablation; EB, embolization; PR, palliative resection; CTx, chemotherapy; RTx, Radiotherapy.

## [18F]FDG-PET-CT and Selective Venous Sampling Improves Localization Diagnostics

After biochemical recurrence in our patient, tumor localization was not possible by various imaging techniques. However, [18F]FDG-PET-CT was able to visualize the unusual localization of abdominal recurrence. In several a case series, [18F]FDG-PET-CT has been shown to effectively localize PC manifestation sites at initial diagnosis, follow-up or recurrence (17). In the current literature, [18F]choline-PET-CT was compared in small series with [18F]FDG-PET-CT. In 2 cases [18F]FDG-PET-CT detected tumor manifestations in the liver and bone lesions in the pelvis, which was missed by [18F]FDG-PET-CT. The authors of that study discussed that the differences in choline and FDG uptake could be the differences in tumor proliferation and differentiation (18). Another case report demonstrated that [18F]FDG-PET-CT and [18F]choline-PET-CT are feasible, in (recurrent) PC (19). In summary, as long as there is no standardized diagnostic work-up especially in recurrent PC, whole body functional imaging should be considered for detecting uncommon tumor localization and to avoid repeated cervical surgery.

Furthermore, in our case the selective venous sampling was additionally performed to confirm this uncommon localization. Therefore, selective venous sampling was able to support the localization diagnostics and consecutively, the focused surgical approach we then performed.

## Molecular Informed Systemic Treatment

Overall, only few reports have characterized the molecular landscape of PC. Using exome sequencing, Yu et al. (20) in a series of seven cases found an average of 51 somatic variants. This is in stark contrast to the findings in our patient who had high tumor mutational burden (TMBh). TMBh is increasingly recognized as a marker of response to immunotherapy (21) and related to the accumulation of tumoral neoantigens which increase the likelihood of response to inhibition of immune checkpoint molecules.

Molecular guided systemic treatment with pembrolizumab led to stable disease as best response on imaging and a marked clinical and biochemical benefit with a stabilization of serum calcium and drop of PTH for 9 months. Our case demonstrates that in rare tumors, molecular analysis may be useful to detect druggable targets. So far, no case of successful treatment of PC with immune checkpoint inhibitors has been described.

## Targeted Surgical Resection Permitted Long-Term Disease Control

After the first two resections of parathyroid metastases, calcium normalization has been achieved. The patient was subsequently clinically and biochemically disease free for 9 months (at last observation 10/2020). Repeated surgery was able to control serum calcium supporting that every effort should be made to localize the disease and evaluate resectability (16, 17). It must be noted that our previous case series suggests that more extended primary surgery (parathyroidectomy and hemithyroidectomy) may be beneficial in lowering the rate of recurrence in general although it is unclear whether this is also true for the very rare distant metastases (7).

## Patients Perspective

Our patient has been alive with a “chronic” disease over a long duration which required medical treatment for long periods of time. Uncertainty about prognosis, eventual diagnostic success and risk of surgery have contributed to the disease burden. The present outcome with biochemical remission has significantly improved the well-being of the patient.

## Conclusion

Because manifestation of recurrent PC outside of the neck and chest is possible, whole body imaging for tumor localization is useful to allow for focused repeat surgery. The course of the disease in our patient with a recurrence after 12 years after primary surgery suggests that the tumor evaded immune recognition at a certain time point during disease progression which may also explain the unusual systemic spread of the disease. It is tempting to speculate that pembrolizumab treatment inhibited further progression of disease rendering surgical removal effective in a neo-adjuvant manner. A multimodal diagnostic approach and therapy in an interdisciplinary setting is needed for patients with rare endocrine tumors.

## Data Availability Statement

The original contributions presented in the study are included in the article/**Supplementary Material**. Further inquiries can be directed to the corresponding author.

## Ethics Statement

Written informed consent was obtained from the individual(s) for the publication of any potentially identifiable images or data included in this article.

## Author Contributions

CL, NS, and MK designed the study. AB and RK performed diagnostic imaging and procedures. JR and AW performed surgical procedures. CG supervised all surgical procedures. SK examined all histopathological samples. AS, DH, and SF performed whole exome sequencing. CL, CF, and MK provided data. CL and MK analyzed the data. CL, MF, NS, CG, AS, DH, SF, and MK interpreted the data. CL and MK wrote a manuscript draft. All authors contributed to the article and approved the submitted version.

## Funding

This publication was supported by the Open Access Publication Fund of the University of Wuerzburg.

## Conflict of Interest

The authors declare that the research was conducted in the absence of any commercial or financial relationships that could be construed as a potential conflict of interest.

## References

[1] Ryhanen EM Leijon H Metso S Eloranta E Korsoff P Ahtiainen P. A nationwide study on parathyroid carcinoma. Acta Oncol (2017) 56(7):991–1003. 10.1080/0284186X.2017.1306103 28362521

[2] Obara T Fujimoto Y. Diagnosis and treatment of patients with parathyroid carcinoma: an update and review. World J Surg (1991) 15(6):738–44. 10.1007/BF01665308 1767540

[3] Lee PK Jarosek SL Virnig BA Evasovich M Tuttle TM. Trends in the incidence and treatment of parathyroid cancer in the United States. Cancer (2007) 109(9):1736–41. 10.1002/cncr.22599 17372919

[4] Cetani F Pardi E Marcocci C. Parathyroid Carcinoma. Front Horm Res (2019) 51:63–76. 10.1159/000491039 30641523

[5] Harari A Waring A Fernandez-Ranvier G Hwang J Suh I Mitmaker E. Parathyroid carcinoma: a 43-year outcome and survival analysis. J Clin Endocrinol Metab (2011) 96(12):3679–86. 10.1210/jc.2011-1571 21937626

[6] Witteveen JE Haak HR Kievit J Morreau H Romijn JA Hamdy MA. Challenges and pitfalls in the management of parathyroid carcinoma: 17-year follow-up of a case and review of the literature. Horm Cancer (2010) 1(4):205–14. 10.1007/s12672-010-0042-6 PMC3000473 21258429

[7] Lenschow C Schragle S Kircher S Lorenz K Machens A Dralle H. Clinical Presentation, Treatment, and Outcome of Parathyroid Carcinoma: Results of the NEKAR Retrospective International Multicenter Study. Ann Surg (2020). 10.2139/ssrn.3541131 32649472

[8] Cetani F Pardi E Marcocci C. Update on parathyroid carcinoma. J Endocrinol Invest (2016) 39(6):595–606. 10.1007/s40618-016-0447-3 27001435

[9] Campenni A Ruggeri RM Sindoni A Giovinazzo S Calbo E Ieni A. Parathyroid carcinoma presenting as normocalcemic hyperparathyroidism. J Bone Miner Metab (2012) 30(3):367–72. 10.1007/s00774-011-0344-y 22246083

[10] Wang P Xue S Wang S Lv Z Meng X Wang G. Clinical characteristics and treatment outcomes of parathyroid carcinoma: A retrospective review of 234 cases. Oncol Lett (2017) 14(6):7276–82. 10.3892/ol.2017.7076 PMC5754841 29344163

[11] Machado NN Wilhelm SM. Parathyroid Cancer: A Review. Cancers (Basel) (2019) 11(11):1676. 10.3390/cancers11111676 PMC6896123 31661917

[12] Manente P Cecchettin M Infantolino D Foscolo G Conte N. Apparently nonfunctioning metastases of parathyroid carcinoma. Tumori (1987) 73(2):191–3. 10.1177/030089168707300218 3576716

[13] Qiu ZL Wu CG Zhu RS Xue YL Luo QY. Unusual case of solitary functioning bone metastasis from a “parathyroid adenoma”: imagiologic diagnosis and treatment with percutaneous vertebroplasty–case report and literature review. J Clin Endocrinol Metab (2013) 98(9):3555–61. 10.1210/jc.2013-2014 23861459

[14] WEEKLY clinicopathological exercises; parathyroid carcinoma with hyperparathyroidism and with metastases to liver. N Engl J Med (1953) 248(10):426–32. 10.1056/NEJM195303052481007 13025711

[15] Lloyd RV Osamura RY Klöppel G Rosai J. WHO classification of tumours of endocrine organs by International Agency for Research. In: Lloyd RV, editor. Cancer, 4th ed, vol. 10 th. World Health Organization (WHO, Genf, Switzerland (2017).

[16] Storvall S Ryhanen E Bensch FV Heiskanen I Kytola S Ebeling T. Recurrent Metastasized Parathyroid Carcinoma-Long-Term Remission After Combined Treatments With Surgery, Radiotherapy, Cinacalcet, Zoledronic Acid, and Temozolomide. JBMR Plus (2019) 3(4):e10114. 10.1002/jbm4.10114 31044184 PMC6478586

[17] Evangelista L Sorgato N Torresan F Boschin IM Pennelli G Saladini G. FDG-PET/CT and parathyroid carcinoma: Review of literature and illustrative case series. World J Clin Oncol (2011) 2(10):348–54. 10.5306/wjco.v2.i10.348 PMC3191327 22022662

[18] Deandreis D Terroir M Al Ghuzlan A Berdelou A Lacroix L Bidault F. (1)(8)Fluorocholine PET/CT in parathyroid carcinoma: a new tool for disease staging? Eur J Nucl Med Mol Imaging (2015) 42(12):1941–2. 10.1007/s00259-015-3130-6 26253272

[19] Hatzl M Roper-Kelmayr JC Fellner FA Gabriel M. 18F-Fluorocholine, 18F-FDG, and 18F-Fluoroethyl Tyrosine PET/CT in Parathyroid Cancer. Clin Nucl Med (2017) 42(6):448–50. 10.1097/RLU.0000000000001652 28394837

[20] Yu W McPherson JR Stevenson M van Eijk R Heng HL Newey P. Whole-exome sequencing studies of parathyroid carcinomas reveal novel PRUNE2 mutations, distinctive mutational spectra related to APOBEC-catalyzed DNA mutagenesis and mutational enrichment in kinases associated with cell migration and invasion. J Clin Endocrinol Metab (2015) 100(2):E360–4. 10.1210/jc.2014-3238 25387265

[21] Passaro A Stenzinger A Peters S. Tumor Mutational Burden as a Pan-cancer Biomarker for Immunotherapy: The Limits and Potential for Convergence. Cancer Cell (2020) 38(5):624–5. 10.1016/j.ccell.2020.10.019 33171127

