# Supplementary material for: Case Report: Abdominal Lymph Node Metastases of Parathyroid Carcinoma: Diagnostic Workup, Molecular Diagnosis, and Clinical Management
Source: Front Endocrinol (Lausanne). 2021 Mar 23;12:643328. doi: 10.3389/fendo.2021.643328 (PMC8021949; doi:10.3389/fendo.2021.643328)
Supplement: Supplementary file 1 [file Table_1.docx]

| **Vein**  **Localization** | **Anatomical Localization** | **PTH [pg/mL]** | | **Ratio PTH vs.**  **V. cava inferior** |
| --- | --- | --- | --- | --- |
|  |  | **Right** | **Left** |  |
| V. jugularis | C1 | 608 | n.a. | 1.04 |
|  | C3 | 626 | n.a. | 1.07 |
|  | C5 | 612 | n.a. | 1.05 |
|  | C7 | 665 | 605 | 1.14 / 1.03 |
| V. thyroidea media |  | 612 | 636 | 1.05 / 1.09 |
|  | Th1 | 639 | n.a. | 1.09 / n.a. |
|  | Th2 | 638 | 614 | 1.09 / 1.05 |
| V. brachiocephalica distal |  | 662 | 649 | 1.13 / 1.11 |
| V. brachiocephalica proximal |  | 659 | 625 | 1.13 / 1.07 |
| V. thyroidea inferior (ima) |  | n.a. | n.a. | n.a. / n.a. |
| V. cava superior |  | 600 |  | 1.03 |
| V. azygos |  | 605 |  | 1.03 |
|  | Right atrial | 676 |  | 1.16 |
| V. hepatica |  | **758** |  | 1.30 |
| V. renalis |  | 462 / 384 |  | 0.79 / 0.66 |
| V. cava inferior |  | 585 |  | 1 |
|  |  |  |  |  |
